# The Relationship Between Staff and Patient Interactions and Patients' Perceptions of Good Quality of Care in Psychiatric Outpatient Services—A Structural Equation Model

**DOI:** 10.1111/scs.70043

**Published:** 2025-06-02

**Authors:** Mikael Rask, Patrik Rytterström, Tabita Sellin, Lars‐Olov Lundqvist, Agneta Schröder, David Brunt

**Affiliations:** ^1^ School of Health and Caring Sciences Linnaeus University Växjö Sweden; ^2^ Division of Nursing Sciences and Reproductive Health, Department of Health, Medicine and Caring Sciences Linköping University Norrköping Sweden; ^3^ Faculty of Medicine and Health, University Health Care Research Center Örebro University Örebro Sweden; ^4^ Department of Nursing, Faculty of Health, Care and Nursing Norwegian University of Science and Technology (NTNU) Gjövik Norway

**Keywords:** psychiatric outpatient care, quality of care, staff–patient interactions, structural equation model

## Abstract

**Background:**

The therapeutic relationship has been identified as essential for ensuring high‐quality care in psychiatric outpatient services, as highlighted in several studies. This study focuses on the content of interactions between patients and staff in psychiatric outpatient care, as well as the quality of care provided, as perceived by the patients.

**Aim:**

The aim of this study is to explore the relationship between these interactions and aspects of the perceived quality of care from the perspective of patients in psychiatric outpatient services.

**Methods:**

A sample of 706 patients from psychiatric outpatient clinics in Sweden completed the Verbal and Social Interaction Outpatient (VSI‐OP) and the Quality in Psychiatric Care—Outpatient (QPC‐OP) instruments. A structural equation model was conducted to explore these associations. The study was approved by the Regional Ethical Review Board in Uppsala, Sweden (Dnr. 2018/186).

**Results:**

The model revealed that the staff showing an interest in the patients' feelings, experiences and behaviour influences the patients' perceived quality of participation through an intricate network of mediator variables, including the staff's ability to establish a relationship, a good encounter, support and a high level of information to the patients. High levels of Participation/Empowerment indicate a high level of quality of care in a psychiatric outpatient context.

**Implication for Practice:**

The presented model provides an understanding of the associations between staff and patient interactions and perceived quality of care and sheds light on important aspects of quality of care from the perspective of the patients.

## 1 Introduction

A common theme of studies focusing on the determinants of good quality in psychiatric care is the therapeutic relationship between patients and staff, which has been referred to as the ‘therapeutic bond with clinicians’ in the literature review by Seed et al. [1]. Aspects of therapeutic relationships were ranked by patients as having the highest importance in care and treatment in a study by Längle et al. [2] and the authors maintained that this was significant for quality of care. Similarly, Killaspy et al. [3] have included therapeutic environment as one of the domains in their instrument for assessing quality of care in psychiatric settings.

In the specific outpatient psychiatric context, Johansson and Eklund [4] have similarly highlighted the importance of nursing staff/therapists in creating a helping encounter/relationship. The patients in their study perceived good care as being constituted by the quality of the helping relationship, which included warmth, empathy, understanding, time and the therapist's ability to enter into the patient's feelings and understand his/her problems and situation. Furthermore, a scoping review of research literature about interventions in community mental health services, containing both quantitative and qualitative methodologies, revealed the alliance between a patient and a member of staff as the one common factor in the 60 publications identified [5]. Several terms have been used in the literature referred to above to denote the therapeutic relationship. We maintain that the relationship can be seen as more oriented toward caring than curing as expressed by Montgomery and Webster [6] and can be therapeutic in its own right with less of a focus on symptoms and problems [7].

Healthcare professionals in psychiatric outpatient services meet patients regularly to provide various types of treatment: counselling, talking therapies, practical support, etc. To be able to deliver good quality of care containing these treatment strategies, a helping/therapeutic relationship with its related concepts of alliance and encounter is an essential factor as indicated above. Luther et al. [8] found that patients in psychiatric outpatient services perceived a high level of quality of care if the staff–patient relationship had, among other factors, a high level of therapeutic alliance. The core elements of the staff/patient interactions generating good quality care thus appear to include the establishment of a helping relationship between the two, the qualities of this relationship and the staff taking an interest in the patients' feelings and circumstances and providing support. This is similar to the findings in the study by Shattell et al. [9], where patients' experiences of the therapeutic relationship were expressed in three themes: ‘relate to me’, ‘know me as a person’ and ‘get to the solution’.

The essential role of the therapeutic relationship for creating good quality of care in psychiatric outpatient services has been demonstrated above and research has revealed a number of attributes and characteristics of this relationship. These have, however, been described in broader terms, for example, warmth, empathy and understanding problems. No study has, to our knowledge, focused more directly on the interactions that occur in the relationship between staff and patients and in which way these are perceived as contributing to good quality of care by the patients. The focus of this study is on the interactions between patients and staff and the quality of the care provided in a psychiatric outpatient context from the patients' perspective. The aim of this study is thus to explore the relationship between staff and patient interactions and aspects of the perceived quality of care from the perspective of patients in psychiatric outpatient services.

## 2 Method

The exploration of the interactions between patients and staff was performed with the VSI‐OP questionnaire and of quality of care with the QPC‐OP questionnaire. The further exploration of the relationship between these was performed with a structural equation model (SEM).

### 2.1 Participants and Procedure

A sample of 706 patients from 15 psychiatric outpatient clinics in three regions in Sweden participated in the study. The mean age of the patients was 36.3 years, ranging from 18 to 87 years of age. A majority of the participants (70%) were women. Anxiety disorder (*n* = 202, 28.6%), neuropsychiatric disorder (*n* = 169, 23.9%) and depression (*n* = 145, 20.5%) were the most commonly self‐reported diagnoses, and 174 patients did not report any diagnosis (Table 1). The inclusion criteria for the patients were: being able to read and understand Swedish, being treated in a psychiatric outpatient clinic, ≥ 18 years old. The exclusion criteria were dementia or confusion or being assessed by the clinic staff as unable to participate. The participating patients received oral and written information by designated staff and those who gave their oral consent were then asked to complete the questionnaires prior to leaving the clinic. The study was approved by the Regional Ethical Review Board in Uppsala, Sweden (Dnr. 2018/186).

**TABLE 1:** Socio‐demographic and background data of patients in outpatient psychiatric services (*n* = 706).

Age in years	Mean (SD and range)	36.3 (18–87)
Sex *n* (%)	Men	207 (29)
	Women	477 (68)
	Other	4 (0.5)
	Missing	18 (2.5)
Diagnosis *n* (%)^a^	Anxiety disorder	202 (28.6)
	Bipolar disorder	72 (10.2)
	Dependency disorder	5 (0.7)
	Depression	145 (20.5)
	Eating disorder	28 (4.0)
	Neuropsychiatric disorder	169 (23.9)
	Personality disorder	57 (8.1)
	Psychosis	43 (6.1)
	Missing data	174 (24.6)
Satisfaction with treatment	Mean (SD)	3.12 (0.6)
Want to return to the clinic	Mean (SD)	4.20 (0.9)
Current mental health	Mean (SD)	2.92 (1.1)
Current physical health	Mean (SD)	3.04 (1.0)

^a^ Patients could have more than one diagnosis.

The terms healthcare professionals, staff and nursing staff and therapists have been used in the relevant background literature presented above. There was a wide range of different healthcare professions working at the outpatient clinics in the present study. We have chosen to use the term ‘staff’ for the sake of simplicity in the rest of the manuscript to designate those providing the treatment to the patients at the psychiatric outpatient clinics.

### 2.2 Measures

The outpatient version of the Verbal and Social Interactions questionnaire (VSI‐OP) [10] was used in order to explore the interactions between patients and staff. This questionnaire has been used in various psychiatric settings for measuring the occurrence and importance of caring interactions according to the patients and staff and focuses on relational aspects, the staff's interest in the patient and the staff's help to establish structure and routines [11, 12]. The original Verbal and Social Interactions instrument (VSI‐FP) consisted of 50 items [13] for studying caring interactions in forensic psychiatric settings. The number of items was subsequently reduced to 21, containing three factors [14]. The denominations of the three factors have been rephrased in the current study to reflect the perspective of the participants, the patients. These factors are: ‘To be invited by the staff to establish a relationship’ (shortened to ‘Inviting a relationship’) with six items (e.g., ‘my treatment provider/contact person shows me that he/she is honest’), ‘The staff show interest in my feelings, experiences and behaviour’ (shortened to ‘Showing interest’) with six items (e.g., ‘my treatment provider/contact person talks to me about my feelings’), and ‘Helping me to establish structure and routines in my everyday life’ (shortened to ‘Helping’) with five items (e.g., ‘my treatment provider/contact person describes to me why it is important for me to keep regular sleeping hours’). The exact expressions of each item were modified to conform to the specific context of the clinical care of outpatients. The VSI‐OP patient version has been psychometrically tested with Confirmatory Factor analysis and a further reduction of items led to 17‐item patient version, which demonstrated satisfactory psychometric properties and satisfactory resemblance with the three‐factor structure in the original VSI‐FP version [10]. The 17 items have four response categories organised in a Likert‐like scale from 1 (not at all) to 4 (very high degree). Cronbach's alpha varied from 0.89 to 0.94 for the three factors and for the entire questionnaire alpha was 0.93 in the psychometric test study [10] and in the present study using the same sample.

The QPC‐OP instrument [15], which has been specifically developed to measure the perceived quality of care in this setting, was used in order to study the patients' perceptions of the quality of the psychiatric outpatient care. The original QPC instrument, which was based on patients' perceptions of what constitutes quality in psychiatric care [16, 17, 18], has been further evolved for use in various psychiatric settings. QPC‐OP contains 30 items reflecting eight different dimensions of perceived quality in psychiatric care: *Encounter* with six items (e.g., ‘if I was angry and irritated my treatment provider/contact person was concerned enough to want to know why’), *Participation*/*Empowerment* with three items (e.g., ‘my opinion about what was the correct care and treatment for me was respected’), *Participation*/*Information* with five items (e.g., ‘I got to recognise signs of deterioration in my mental health’), *Discharge* with three items (e.g., ‘I received information about where I could go if I needed help after contact with the clinic came to an end’), *Support* with four items (e.g., ‘my treatment provider/contact person prevented me from harming myself if I had such thoughts’), *Environment* with three items (e.g., ‘I was secure together with my fellow patients in the waiting room’), *Next of kin* with two items (e.g., ‘my next of kin were offered the opportunity to take part in my care and treatment to the extent I wished’) and *Accessibility* with four items (e.g., ‘it was easy to get an appointment with my treatment provider/contact person’). The response categories are organised in a Likert‐like scale with four alternatives from 1 (totally disagree) to 4 (totally agree). The QPC‐OP also contains background questions: age, sex, perceived satisfaction with treatment, if the patient wanted to return to the clinic, perceived current mental health, perceived current physical health and self‐reported diagnosis (Table 1). The QPC‐OP has shown a clear factor structure and good reliability [15]. Cronbach's alpha varied from 0.65 to 0.94 for the eight dimensions and for the entire questionnaire alpha was 0.95. Cronbach's alpha in the present sample varied from 0.69 to 0.94 and for the entire questionnaire alpha was 0.97.

### 2.3 Statistical Analysis

The SPSS 26 software was used for descriptive statistics, frequencies, mean, standard deviation and percentages. Imputation in the case of missing data in the two questionnaires was performed by replacing missing data points, which had 30% or less missing data, by using the Expectation–Maximisation (EM) algorithm [19] in SPSS with the participant's mean item value of each dimension in QPC as well as each factor in VSI. The SEM was conducted with the AMOS 26 software to determine the associations between the Verbal and Social Interactions (VSI) and the aspects of Quality of Psychiatric Care (QPC) in outpatient settings. A *p*‐value of 0.05 was considered significant for the SEM. Non‐significant factors and dimensions were removed stepwise from the model guided by modification indices. Different direct and indirect paths (mediating effects) were tested to determine the relationships between the factors in VSI and QPC to attain a model that demonstrated as good a fit as possible, related to the data. The following values were used for testing the model fit: *χ* ^2^/df (< 3), comparative fit index (CFI) ≥ 0.90 for acceptable fit and ≥ 0.95 for excellent fit, root mean square error of approximation (RMSEA) ≤ 0.08 for adequate fit and < 0.05 as excellent level [20] and standardised root mean square residual (SRMR) ≥ 0.10, considered adequate and > 0.08 as excellent [21].

## 3 Results

The analysis of the responses to the VSI‐OP questionnaire showed that the patients perceived that being invited by the staff to establish a relationship was the most frequently occurring of the factors, followed by the staff showing an interest in their feelings, experiences and behaviour. The dimensions of quality of care rated as having a high level of quality were *Encounter*, *Environment*, *Next of kin* and *Support* (Table 2).

**TABLE 2:** Descriptives of VSI (factors) and QPC (dimensions) (*n* = 706).

VSI		QPC	
VSI factors	Total mean (SD)	QPC dimensions	Total mean (SD)
Inviting a relationship	3.47 (0.6)	Encounter	3.60 (0.6)
Showing interest	2.82 (0.8)	Environment	3.42 (0.7)
Helping	2.03 (0.9)	Next of kin	3.23 (1.0)
		Support	3.24 (0.9)
		Participation empowerment	3.14 (0.8)
		Participation information	3.02 (0.8)
		Accessibility	2.82 (0.9)
		Discharge	2.82 (0.9)

**FIGURE 1:**
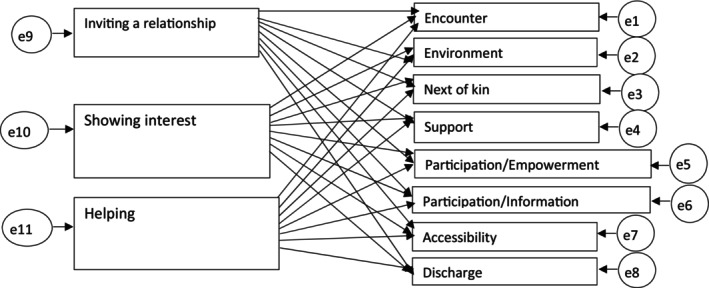
Initial model.

**FIGURE 2:**
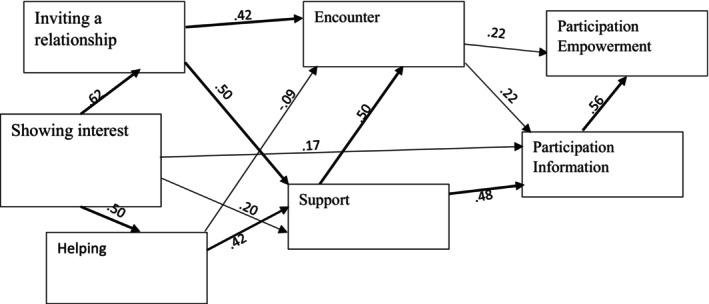
The final model VSI = > QPC outpatient standardised estimates.

The initial model (Figure 1) did not show a satisfactory fit, and as stated above, the non‐significant dimensions were thus removed. The three VSI factors *Showing interest*, *Inviting a relationship* and *Helping* and the four QPC dimensions *Encounter*, *Support*, *Participation*/*Information* and *Participation*/*Empowerment* were retained in the revised model (Figure 2). As the aim of the study was to explore the relationship between staff and patient interactions and aspects of the perceived quality of care, we used the SEM in an explorative manner. After systematically testing different direct and indirect effects, the goodness‐of‐fit for the final model was acceptable (*χ* ^2^ = 14.471; df = 8; *p*‐level = 0.070 CFI = 0.998; RMSEA = 0.034 and SRMR = 0.016).

The model showed that the factor ‘Showing interest’ directly influences the other two factors ‘Inviting a relationship’ and ‘Helping’ (Figure 2), which in turn influence *Encounter* and *Support* (Figure 2). The factor ‘Showing interest’ also influences *Support* and *Participation*/*Information. Encounter* and *Support* served mutually and separately as mediators to increase the level of *Participation*/*Information*. Additionally, *Participation*/*Information* served as a mediator from ‘Showing interest’, *Encounter* and *Support* to increase the level of perceived *Participation*/*Empowerment*. The factor ‘Showing interest’ affects the patients' perceived quality of participation through an intricate network of mediator variables, including the staff's ability to establish a relationship, a good encounter, support and a high level of information to the patients.

The direct, indirect and total effects on the quality of psychiatric outpatient care *Participation*/*Empowerment* were estimated in the final mediation model (Table 3), which revealed that the factor ‘Showing interest’ had a significant and high total effect on *Participation*/*Empowerment* as well as the factor ‘Inviting a relationship’. The factor ‘Helping’ had a significant but lower and less significant total effect on *Participation*/*Empowerment*. Furthermore, *Support*, *Encounter* as well as *Participation*/*Information* also had a significant and high total effect on *Participation*/*Empowerment*. *Support*, *Encounter* and the factor ‘Inviting a relationship’ had strong indirect/mediating effects on *Participation*/*Empowerment* in the model. The factor ‘Helping’ had a significant but lower and less significant indirect effect. The only aspects that had a direct significant effect on *Participation*/*Empowerment* were *Encounter* and *Participation*/*Information*.

**TABLE 3:** Direct, indirect and total effects on quality of psychiatric outpatient care participation empowerment in the final mediation model.

	Direct	Indirect	Total
Showing interest			0.427**
Inviting a relationship		0.444**	0.367**
Helping		0.026*	0.030*
Support		0.443**	0.443**
Encounter	0.277**	0.346**	0.346**
Participation information	0.557**		0.563**
*Model fit*			
Chi‐squared; degree of freedom; *p*‐value	*χ* ^2^ = 14.471; df = 8; *p* = 0.070		
RMSEA (90% CI)	0.034 (0.000; 0.061)		
CFI	0.998		
SRMR	0.016		
Model *R* ^2^	0.533		

*Note:* Coefficients are standardised.

Abbreviations: CFI, comparative fit index; RMSEA, root mean square error of approximation; SRMR, standardised root mean squared residual.

*p < 0.05 and **p < 0.01.

## 4 Discussion

The aim of this study was to explore the relationship between staff and patient interactions and aspects of the perceived quality of care from the perspective of patients in outpatient psychiatric care. The patients rated that being invited to establish a relationship was the most frequently occurring of the staff's verbal and social interactions, followed by the staff's interest in the patients' feelings, experiences and behaviour and on helping them to establish structure and routines in their everyday life. The final fit in the structural equation model revealed that the factor ‘Showing interest’ directly influenced the other two VSI factors and two of the QPC dimensions, and through pathways of mediating factors led to influence the level of perceived *Participation*/*Empowerment*. *Encounter* and *Participation*/*Information* were the only aspects that had a direct effect on *Participation*/*Empowerment*.

### 4.1 Aspects of the Staff–Patient Interactions of Importance for Quality of Care

All the three VSI‐factors had a direct influence on one or more of the QPC‐dimensions and an indirect influence on *Participation*/*Empowerment* according to the model. These variables thus had significant roles in the model in which the variable ‘Showing interest’ directly influenced the other two VSI‐factors. This finding is significant in the light of the research into the determinants of good quality in psychiatric care, where the presence of a therapeutic relationship between patients and staff, also termed the therapeutic bond or the therapeutic alliance, is a common feature [1, 2, 4, 5]. It would be reasonable, based on the findings in these studies, for the variable ‘Inviting a relationship’ to be the one directly influencing the other two VSI‐factors in the model. The importance of ‘Inviting a relationship’ can be seen as it was rated the highest of the VSI‐factors by the patients in the present study and influenced both *Encounter* and *Support*. This observation aligns closely with the patterns identified in the literature above, reinforcing the idea that the initiation of a therapeutic relationship by the staff in outpatient psychiatric services significantly shapes the patient's perception of receiving adequate support and a personal encounter.

However, despite the well‐documented importance of the relationship between patients and staff in determining the quality of outpatient psychiatric care as referred to above, ‘Showing interest’ by the staff was found to be an antecedent to ‘Inviting a relationship’. This concurs with the views of the patients in the study by Johannson and Eklund in an outpatient setting where the therapist's ability to enter into the patient's feelings and understand his/her problems was seen as important for good quality of care. Rytterström et al. [22], in a study of encounters in a forensic psychiatric context, report on significant encounters as a phenomenon that can underpin the development of a relationship between staff and patients, and for these encounters to occur, it is essential that both parties are open to sharing experiences and personal needs to establish a significant encounter. Moreover, Dahlberg [23] points out that a caring relationship is an essential element in the staff–patient relationship, but further maintains that the staff need to have a listening attitude and are prepared to encourage the patient to share his/her feelings and thoughts about what is troubling them in order to be able to establish a healing staff‐patient conversation. The patients' perception of the staff's interest in their feelings, experiences and behaviour in the present study constitutes the initial approach from the staff. The healthcare professionals in psychiatric outpatient services may well feel that they already show such an interest, but its significance as a precursor to establishing a relationship, as shown in the model, is perhaps less well recognised. This has to our knowledge not been previously reported in this context and is thus a significant finding indicating an essential and fundamental element as the starting point for achieving a high level of quality of care.

The third aspect of the staff–patient interactions, ‘Helping’, appears to have a more inferior role with the weakest indirect influence on the *Participation*/*Empowerment*, and weak correlations with *Encounter* and *Support*, *which* also confirm this picture. This variable was the least frequently occurring of the VSI factors in the present study in the psychiatric outpatient care context and was rated at similarly low levels in a supported housing context [11] and in a forensic psychiatric context [12] thus confirming this factor's less prominent role in the eyes of the patients.

### 4.2 Aspects That Contribute to the Level of Participation/Empowerment

The *Encounter* dimension was rated as having the highest quality of care by the patients in this study, which concurs with results from previous studies using the QPC instrument in other psychiatric contexts, such as inpatient care [18], outpatient care [15], and ordinary housing with housing support [24]. The *Encounter* dimension in the QPC and the factor ‘Inviting a relationship’ in the VSI are strongly correlated, most likely due to both including items concerning the staff‐patient interpersonal relationship. Their significant position in the model and influence on *Participation*/*Empowerment* corroborate the body of evidence presented above concerning the importance of the relational aspect in the quality of care in an outpatient psychiatric context. However, it is notable that *Encounter* is one of the two dimensions to have a direct influence on *Participation*/*Empowerment*, whereas also having an ancillary role as one of the mediators in an intricate network of variables that indirectly influence the level of *Participation*/*Empowerment*. Moreover, Encounter also has a lower total effect on *Participation*/*Information* than the *Support* dimension.

The *Support* dimension, on the other hand, is strongly associated with ‘Inviting a relationship’ and directly influences both *Encounter* and *Participation*/*Information*, indicating a significant mediating role in the effect on *Participation*/*Empowerment*. The items in the *Support* dimension [18] in the QPC‐instrument focus on the staff preventing the patient from hurting him/herself or others and not needing to feel ashamed due to having mental ill health. The nature of these items differs to some extent from a broader perspective on the concept of support that can be found in the literature; for example, Chronister et al. [25] revealed five support categories in a study of family members' perspectives on social supports that are beneficial for people with severe mental illness. An important feature and strength of the QPC‐instrument is that it is based on research into patients' perspectives on the quality of care [17]. The analysis in the present study reveals that the patients in psychiatric outpatient care perceive the importance of the *Support* dimension with the items presented above. *Support* has strong associations with two of the VSI‐factors and two of the QPC‐dimensions and can be seen to exert a mediating and indirect effect in the model.

The two *Participation* dimensions, *Information* and *Empowerment*, were previously combined in one *Participation* dimension in the original inpatient version of the QPC [17]. They have since been shown to be two separate aspects in outpatient care [15] and can be seen in this model as having two distinctly different roles, with the *Participation*/*Information* dimension being the mediator with the strongest direct effect on *Participation*/*Empowerment*. The *Participation*/*Information* dimension includes items concerning recognising signs of deterioration, information given in a way that can be understood, knowledge about mental illness and information about treatment alternatives. These are all essential aspects of information that patients need in order to make informed decisions and be empowered, which is corroborated by the WHO in their statement [26] that decision‐making does not occur in a vacuum and that decisions can only be made when the individuals have sufficient information to make informed decisions. The significant role of information is further endorsed in the model for shared decision‐making as presented by Elwyn et al. [27, 28], where one of the prerequisites is the provision of complete and adequate information about treatment alternatives, and furthermore in a proposed extension to this model by Grim et al. [29] concerning information as a decisional need in a preparatory stage of shared decision‐making. This important role of information as a precursor for the promotion of empowerment is also evident in the essential mediating role of *Participation*/*Information* in the model in this present study.

### 4.3 Empowerment—A Core Aspect of Quality of Care

*Participation*/*Empowerment* is shown to be the final outcome measure in the model, indicating its specific role in the patient perception of quality of care in a psychiatric outpatient context. The items in this dimension: ‘Influence over my care’, ‘My view of the right care is respected’ and ‘Take part in decision‐making about my care’, constitute core elements of the concept of empowerment [30], which has also been identified as a key aspect of recovery‐oriented mental health care [31]. Moreover, the provision of empowerment‐oriented care services has been shown to be more effective than a focus on global function improvement in recovery for patients with schizophrenia [32], thus demonstrating the quality‐of‐care aspects of empowerment. Linhorst [33] maintains that empowerment can be facilitated by the role of staff and others close to the patient in providing supportive relationships and networks, resources and the opportunities for active participation in decision‐making. Elements of this crucial supportive role can be identified in the five variables (‘Inviting a relationship’, ‘Helping’, *Encounter*, *Support* and *Participation*/*Information*) constituting the mediators affecting the level of *Participation*/*Empowerment* in the proposed model. Furthermore, Linhorst and Eckert [34] have described the conditions necessary for empowering people with severe mental illness, where support and information to make informed decisions are among the external conditions the authors describe as necessary for meaningful participation to occur. These two conditions can also be seen to have significant mediating roles in the model where *Support* is influenced by all three of the aspects of the staff–patient interactions and influences *Participation*/*Information*, which in turn exerts the greatest influence on *Participation*/*Empowerment*.

### 4.4 Aspects Not Contributing to the Level of Participation/Empowerment

Four of the QPC dimensions were not significantly correlated to achieve an acceptable model fit and were thus removed. It is important, however, to also discuss these considering their significant role in the QPC instrument. *Discharge* had the lowest ratings for quality of care in the present study, as also found in the initial study using the QPC‐OP instrument [35]. It is reasonable to assume that *Discharge*, with its focus on treatment conclusion and the future, may be of less significance for patients in terms of quality in an outpatient context where treatment periods are often long‐term and thus constitute a less influential variable in the model. The *Environment* dimension was rated highly by the patients in the present study, which has also been found in previous research [35, 36]. The therapeutic effects of the environment in a psychiatric setting are well documented [37], but this aspect does not appear to have an important role in the current model. The nature of the items in the *Environment* dimension focusing on the patients' sense of security, which could be said to have a background quality, may be a reason for this. The nature of the aspects of the quality of care, which were included in the model, had on the other hand a greater emphasis on the actions of the staff and their interactions with the patients.

The *Accessibility* dimension, with a focus on how easy it was for the patient to access the clinic, had similarly low ratings as those for the *Discharge* dimension, and can also be said to have a background quality. The *Next of Kin* dimension, which was highly rated for quality of care in the present study as well as in other studies using the QPC‐OP instrument [35, 36], was also removed from the model. This may well be due to the predominantly staff‐patient focus in the aspects in the model with thus a greater emphasis on in‐house activities while the next of kin, despite their decisive role in the care for the patients [17] can be said to have more of a peripheral role in the specific psychiatric outpatient context. The nature of these four dimensions with a background or more peripheral focus thus appears not to exert a sufficient direct or indirect influence on the *Participation*/*Empowerment* dimension in the model.

## 5 Discussion of Method

Using a structural equation model was a strength in the study as it facilitated the exploration of the complex pattern of relationships between the factors/dimensions in the VSI‐OP and QPC‐OP instruments. The model fit indices generated in the SEM provided a suitable measure for investigating how well the model fitted with the data. Moreover, the relatively large sample size was significant for the detection of possible associations between the factors/dimensions. A potential weakness could be that we were unable to present details of the external loss due to the lack of complete data on the participants who had been asked to participate in the study but had declined to do so. This reduces the possibilities for arguing strongly for the generalisability of the model. Furthermore, this study has a cross‐sectional nature, and the results can thus not show causal relationships between the included variables but instead provide a picture that could guide staff in outpatient services in their encounters with the patients to enhance the quality of the care provided. Longitudinal studies are needed in order to be able to draw further conclusions about causal relationships between what the staff can do to enhance the quality of the mental health care provision. To further strengthen the validity of the model, the study needs to be replicated in outpatient settings. Another approach could be to use other questionnaires focusing on the patients' perception of the staff's interactions with them and how they perceive the quality of the care provided.

## 6 Implications for Practice

The model presented in the study sheds light on what the staff in outpatient psychiatric care can do to increase the patients' perceived quality of the care they receive. First, the significance of the staff showing an interest in the patient's feelings, experiences and behaviour in establishing a staff–patient relationship indicates a need for staff to pay greater attention to the way they conduct their initial approach to the patients and put a greater focus on showing this type of interest. Second, the two dimensions focusing on *Participation* have prominent roles in the model and are essential elements of good quality of care according to the patients. A high level of *Participation*/*Empowerment* indicated a high level of quality of care in a psychiatric outpatient context and *Participation*/*Information* had the strongest direct effect on *Participation*/*Empowerment*. Strategies to increase the patients' participation in their care, for example, by providing the patients with sufficient information for them to be able to make informed decisions, are important for their empowerment and thus the level of the quality of care. Finally, the structural equation model is based on the perceptions of the patients and how they view their interactions with staff and the dimensions of quality of care, which should be a benchmark for rating quality of care.

## Author Contributions

M.R. conceptualization, formal methodology, writing – original draft, writing – review and editing. P.R. conceptualization, writing – review and editing. T.S. conceptualization, writing – review and editing. L.‐O.L. conceptualization, formal methodology, writing – review and editing. A.S. conceptualization, writing – review and editing. D.B. conceptualization, writing – original draft, writing – review and editing.

## Ethics Statement

The study was approved by the Regional Ethical Review Board in Uppsala, Sweden (Dnr. 2018/186).

## Conflicts of Interest

The authors declare no conflicts of interest.

## Data Availability

The data that support the findings of this study are available from the corresponding author upon reasonable request.
